# Mouse gastric tumor models with prostaglandin E_2 _pathway activation show similar gene expression profiles to intestinal-type human gastric cancer

**DOI:** 10.1186/1471-2164-10-615

**Published:** 2009-12-17

**Authors:** Hiraku Itadani, Hiroko Oshima, Masanobu Oshima, Hidehito Kotani

**Affiliations:** 1 Oncology Research Department, Tsukuba Research Institute, Banyu Pharmaceutical Co., Ltd, Tsukuba, Japan; 2 Division of Genetics, Cancer Research Institute, Kanazawa University, Kanazawa, Japan; 3 Presidents's Office and Business Development, Banyu Pharmaceutical Co., Ltd, Tokyo, Japan

## Abstract

**Background:**

Gastric cancers are generally classified into better differentiated intestinal-type tumor and poorly differentiated diffuse-type one according to Lauren's histological categorization. Although induction of prostaglandin E_2 _pathway promotes gastric tumors in mice in cooperation with deregulated Wnt or BMP signalings, it has remained unresolved whether the gastric tumor mouse models recapitulate either of human gastric cancer type. This study assessed the similarity in expression profiling between gastric tumors of transgenic mice and various tissues of human cancers to find best-fit human tumors for the transgenic mice models.

**Results:**

Global expression profiling initially found gastric tumors from *COX-2*/*mPGES-1 *(C2mE)-related transgenic mice (*K19-C2mE*, *K19-Wnt1/C2mE*, and *K19-Nog/C2mE*) resembled gastric cancers among the several tissues of human cancers including colon, breast, lung and gastric tumors. Next, classification of the C2mE-related transgenic mice by a gene signature to distinguish human intestinal- and diffuse-type tumors showed C2mE-related transgenic mice were more similar to intestinal-type compared with diffuse one. We finally revealed that induction of Wnt pathway cooperating with the prostaglandin E_2 _pathway in mice (*K19-Wnt1/C2mE *mice) further reproduce features of human gastric intestinal-type tumors.

**Conclusion:**

We demonstrated that C2mE-related transgenic mice show significant similarity to intestinal-type gastric cancer when analyzed by global expression profiling. These results suggest that the C2mE-related transgenic mice, especially *K19-Wnt1/C2mE *mice, serve as a best-fit model to study molecular mechanism underlying the tumorigenesis of human gastric intestinal-type cancers.

## Background

Gastric cancers are classically categorized into intestinal type and diffuse type based on Lauren's histological classification [1]. Intestinal-type gastric cancers are characterized by better differentiated, cohesive and glandular-like cell groups. The intestinal type is progressed through multiple steps beginning with atrophic gastritis that is followed by intestinal metaplasia, dysplasia and carcinoma [2,3]. Diffuse type corresponds to poorly differentiated, infiltrating and non-cohesive tumor cells. Although diffuse type is not characterized by the multiple proceeding steps, this shows more metastatic phenotype with poorer prognosis.

Several genetic alterations are more frequently observed in either subtype of gastric cancer. Overexpression of *ErbB2 *is selectively found in intestinal-type tumors and may serve as prognostic marker for tumor invasion [4,5]. *ErbB2 *expression level was reported to correlate with lymph node or liver metastasis [6,7]. Significant decrease in the expression of E-cadherin (*CDH1*) has also been described preferentially in diffuse-type gastric cancer ranging from 20% to 90% of frequency [8-10]. The decreased expression of *CDH1 *is caused by LOH or hypermethylation. Interestingly, hereditary diffuse gastric cancer is caused by germline mutations of *CDH1 *gene [11,12]. In addition, mutation in adenomatous polyposis coli (*APC*) which activates Wnt/β-catenin pathway is predominantly found in intestinal-type gastric cancer [13]. Cyclooxygenase-2 (*COX-2*) that is one of the crucial enzymes to synthesize prostaglandin E_2 _is highly up-regulated in intestinal-type cancers compared with diffuse-type ones [14]. These genetic alterations could be used as a hallmark of each type of gastric cancer as well as the histological features.

Genome-wide mRNA expression profiles have identified gene signatures to distinguish intestinal- and diffuse-type gastric cancers. Boussioutas *et al*. [15] reported that the gene signature distinctive for intestinal type exhibits the up-regulation of proliferation markers related to DNA replication, spindle assembly and chromosome segregation. Down-regulated genes in the signature are associated with epithelial differentiation. Jinawath *et al*. [16] also developed another gene signature that is differentially expressed between intestinal-type and diffuse-type cancers with Japanese gastric tumor samples. The intestinal-type signature represented enhancement of cell cycle progression, while the genes associate with extracellular-matrix (ECM) are deregulated in the diffuse type signature. These signatures could provide opportunities of developing biomarkers to diagnose/distinguish the two types in both clinical and preclinical researches.

Transgenic mice that develop gastric tumors present suitable models to decipher gastric tumorigenesis, and identify novel therapeutic targets. We have previously developed several transgenic mice in which prostaglandin E_2 _production pathway is highly activated specifically in gastric mucosa. *K19-C2mE *mice expressing *COX-2 *and microsomal prostaglandin E synthase-1 (*mPGES-1*) develop inflammation-associated hyperplasia [17]. This was mediated through the recruitment of mucosal macrophages. By crossing the *K19-C2mE *mice with *K19-Wnt1 *mice, cooperative effect of Wnt1 and PGE_2 _on gastric tumorigenesis was investigated. The *K19-Wnt1/C2mE *mice led to the development of dysplastic gastric adenocarcinoma signifying the importance of the Wnt pathway activation to keep the progenitor cells undifferentiated [18]. To examine the additional effect of the suppression of BMP pathway on the prostaglandin E_2 _activation, the compound mice of *K19-Nog/C2mE *were established. The *K19-Nog/C2mE *mice cause the development of gastric hamartomas that are morphologically similar to juvenile polyposis (JP) [19]. Although the detailed histological and hypothesis-based molecular analysis implicated the pivotal role of prostaglandin E_2_, Wnt and Nog pathway respectively in gastric tumorigenesis, it remains elusive whether the K19-C2mE and its compound transgenic mice show similarity to intestinal type or diffuse type of human gastric cancers when analyzed by non-biased global expression profile.

In order to identify which types of human gastric tumors (intestinal or diffuse type) the C2mE-related mice are more similar to, we compared expression profile of the two types of human gastric cancer with those of *K19-C2mE*, *K19-Wnt1/C2mE*, and *K19-Nog/C2mE *transgenic mice.

## Results

### Overall gene expression profiles of transgenic animals

We have previously developed several types of transgenic mice in which prostaglandin E_2 _pathway is activated. *K19-C2mE *mice expressing *COX-2 *and *mPGES-1 *induce hyperplasic gastric tumors. *K19-Wnt1/C2mE *mice in which both Wnt and prostaglandin E_2 _pathways are activated cause dysplastic gastric tumors. *K19-Nog/C2mE *mice expressing noggin as well as C2mE develop gastric hamartomas. To provide insight into the molecular mechanism of gastric tumorigenesis, gastric tissues from the transgenic mice and wild-type mice were subject to microarray analysis. Using the Affymetrix GeneChip system, mRNA expression levels were measured for 45,037 probe sets, which represent 21,066 Entrez genes and 5,324 other sequences. Increased expression of introduced gene in each transgenic mouse was observed as reported previously [17-19].

**Figure 1:**
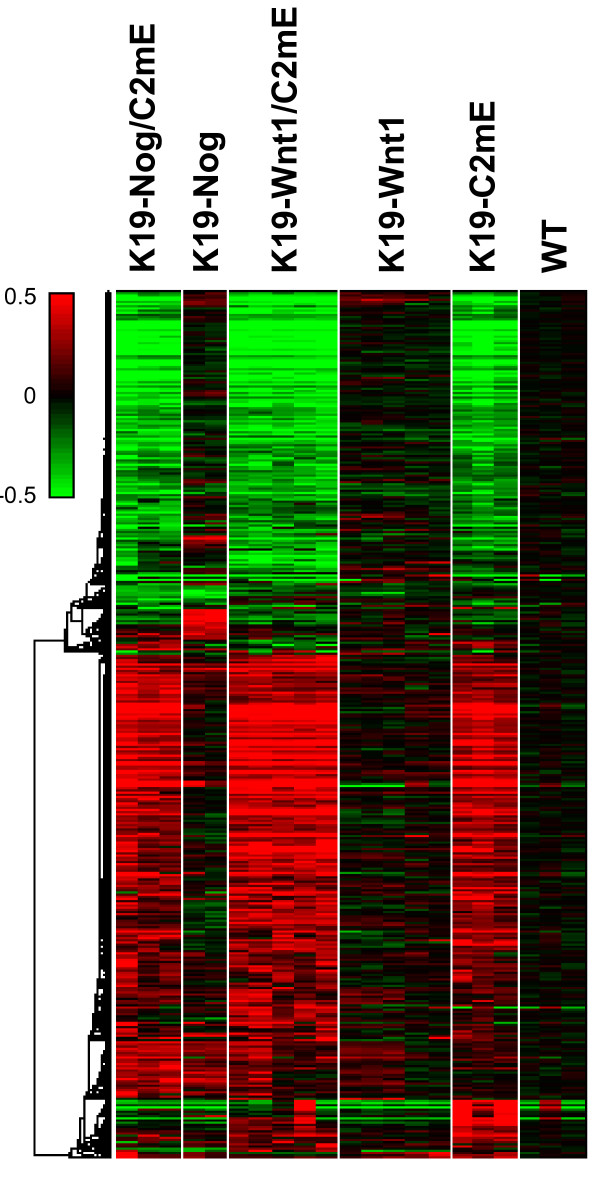
**Genome-scale expression pattern of transgenic mice showing major changes are caused by PGE_2 _induction**. Clustered in rows are 5,440 probe sets selected by fold change threshold of 2 or greater to the average of wild-type and a ratio p-value of 0.01 or less, and columns are mouse gastric samples grouped by genotype. Genotypes are shown on the top of the heatmap. The red-green color scale represents log10 ratio to the average of wild-type samples, as shown in a color bar on top left: red color indicates the gene is up-regulated in the sample, and green indicates down-regulated. WT: wild-type.

Genome-scale overview of the microarray data revealed that expression changes in the three tumor models of *K19-C2mE*, *K19-Wnt1/C2mE *and *K19-Nog/C2mE *were quite similar, whereas overexpression of Wnt1 only or Nog only led to the expression changes in a small portion of genes (Figure 1). This suggests most of expressional changes in the three transgenic mice were caused by the activation of PGE_2 _pathway. Hypergeometric test for gene enrichment showed that the genes involved in wound healing and inflammatory response were significantly condensed with the p-value of 1.5 × 10^-21 ^and 4.2 × 10^-13^, respectively, in the gene set changed by the C2mE induction.

### Classification of mouse tumor models under a human gastric cancer subtype

**Figure 2:**
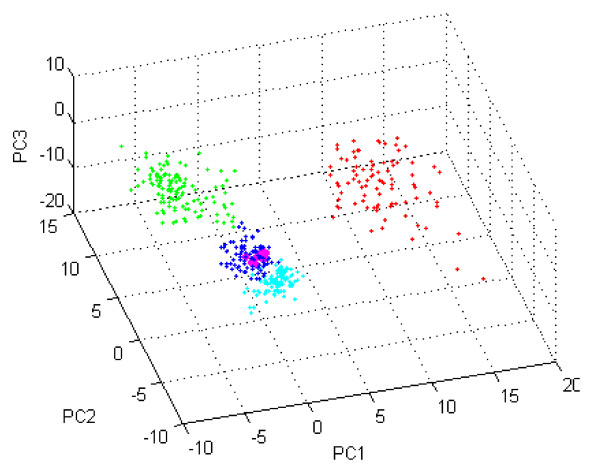
**Overall expression changes in gastric tumors of C2mE-related transgenic mice are most similar to those in human gastric cancers**. *K19-C2mE*, *K19-Wnt1/C2mE*, and *K19-Nog/C2mE *mouse gastric tumors and human gastric (diffuse, intestinal, and mixed type), colon, breast, and lung cancers were plotted by principal component 1 to 3 (PC1 to PC3) calculated using 1,925 genes which were changed by more than two-fold in more than 50 samples of all. The cumulative contribution of the three components was 32%. Dots shown in blue: human gastric cancers; cyan: human colon cancers; red: human lung cancers; green: human breast cancers; magenta: mouse model tumors.

In order to confirm that the mouse gastric tumor models are similar to human gastric cancer, the expression profiles were compared with those of human cancer samples. First, gene expression data of human breast, lung, colon, and gastric tumors were collected from public domain. To estimate similarity between the mouse gastric tumors and the four types of human cancers, supervised classification of principal component analysis (PCA) was conducted using 1,925 genes which were changed more than two-fold in more than 50 samples of all human samples. The PCA with the selected genes found that mouse gastric samples from C2mE-related mice were most closely clustered to human gastric cancers among the four tissues examined, indicating the global expression changes in the gastric tumors of the transgenic mice resembled those in human gastric cancers (Figure 2).

Next, in order to examine which subtype of gastric cancer shows cross-species similarity, the mouse tumors were compared with human gastric intestinal-type and diffuse-type cancers on the basis of their expression profiles. Previous expression profiling studies of human gastric tumor samples have identified gene signatures that classify the two types. Intestinal and diffuse types are the two major types of cancer classified on the basis of microscopic morphology [1]. Boussioutas *et al*. [15] showed that proliferation genes were over-expressed in intestinal-type tumors than in diffuse-type tumors; in contrast, extracellular matrix protein genes were up-regulated in diffuse-type compared with intestinal-type tumors. In order to determine which type of human gastric cancer the mouse models are more similar to, we normalized the human data [20] to the average of normal samples, and selected 122 genes which were changed in the opposite direction in intestinal type and diffuse type [see Additional file 1], to classify intestinal and diffuse types by using the normalized data. The false discovery rate was estimated to be 2.4%. The accuracy of class prediction using this gene set was estimated to be 85% by leave-one-out cross-validation of human samples. We also examined whether this gene set can be used to correctly classify another gastric cancer data set [15]. The test data set included 22 intestinal-type, 35 diffuse-type, and ten normal samples, and was normalized to the average of all normal samples. The error rate was 25% in total, and 29% and 18% in diffuse- and intestinal-type cancers, respectively.

**Figure 3:**
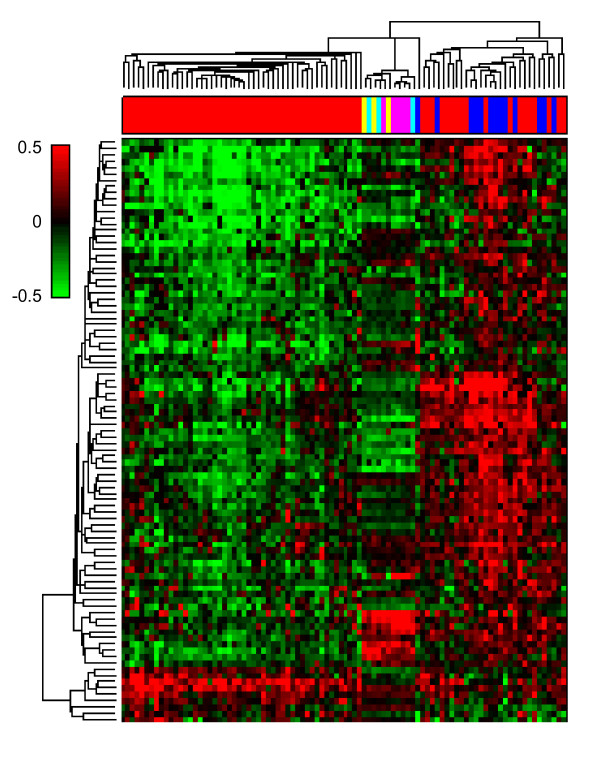
**Expression profiles of C2mE-related gastric tumors are clustered to human intestinal-type gastric cancers**. Clustered in rows are 93 genes which met p-value less than 0.001 and opposite change direction between intestinal-type and diffuse-type human gastric cancers, and clustered in columns are human and mouse gastric tumors. As a distance measure, cosine correlation was used. Linkage method for clustering was average linkage. Samples shown in red: human intestinal type gastric cancers; blue: human diffuse type; yellow: *K19-C2mE *mice; magenta: *K19-Wnt1/C2mE*: cyan: *K19-Nog/C2mE*. The red-green color scale represents log10 ratio to the average of wild-type or normal samples, as shown in a color bar on top left.

To compare the expression patterns of the signature genes in mouse tumors to those in human gastric cancers, hierarchical clustering analysis was performed with mouse gastric data and human intestinal- and diffuse-type data sets. The expression pattern of our modified signature genes for distinguishing intestinal- and diffuse-type gastric cancers revealed that the gastric tumors from C2mE-related transgenic mice were more similar to intestinal-type human gastric cancers than to diffuse-type human gastric cancers (Figure 3). By linear discriminant analysis, all C2mE-related gastric tumors except one K19-Wnt1/C2mE sample were classified as intestinal-type tumors.

### Expression pattern of the genes frequently deregulated in human gastric cancer in a subtype specific manner

It is known that amplification or overexpression of some genes are found in a subtype-specific manner. E-cadherin gene mutations or loss are specifically found in diffuse-type gastric cancer [11,12]. In contrast, amplification of *ErbB2 *gene is observed only in intestinal type, and not reported in diffuse type [6,7]. LOH of deleted in colorectal carcinoma (*DCC*) is predominantly observed in about half of intestinal-type [21,22]. Expression levels of the three genes were compared between mice and human gastric cancer types (Table 1). *CDH1 *expression was significantly decreased in human diffuse type but not in intestinal type as expected. In the three transgenic mice, *Cdh1 *gene was not decreased in any of transgenic mice compared with wild-type, inferring that one of the most characteristic changes in human diffuse type gastric cancer was not observed in the mouse models. Up-regulation of *ErbB2 *was observed in human intestinal-type microarray data, and also in our mouse data. *DCC *expression was reduced in human intestinal-type as expected, while the reduction of the gene was observed in the mice model, especially in *K19-Wnt1/C2mE *mice. The expressions of the three genes defining the tissue-type of the human gastric cancer also support the idea that the mouse models are more similar to intestinal-type human cancer.

**Table 1:** Expression changes of subtype-specific genes in mouse and human gastric tumors.

	Mouse			Human	
		
	C2mE	Wnt1/C2mE	Nog/C2mE	Diffuse	Intestinal
CDH1	1.13*	1.00	1.10	0.43*	1.09
ErbB2	1.37*	1.43*	1.25*	0.92	1.37*
DCC	0.91	0.85*	0.94	0.98	0.71*

Expression values are shown in average log ratios (base 10) to wild-type or normal samples. Asterisk indicates t-test p-value < 0.05.

### Difference among PGE_2 _pathway-activated mouse models

**Figure 4:**
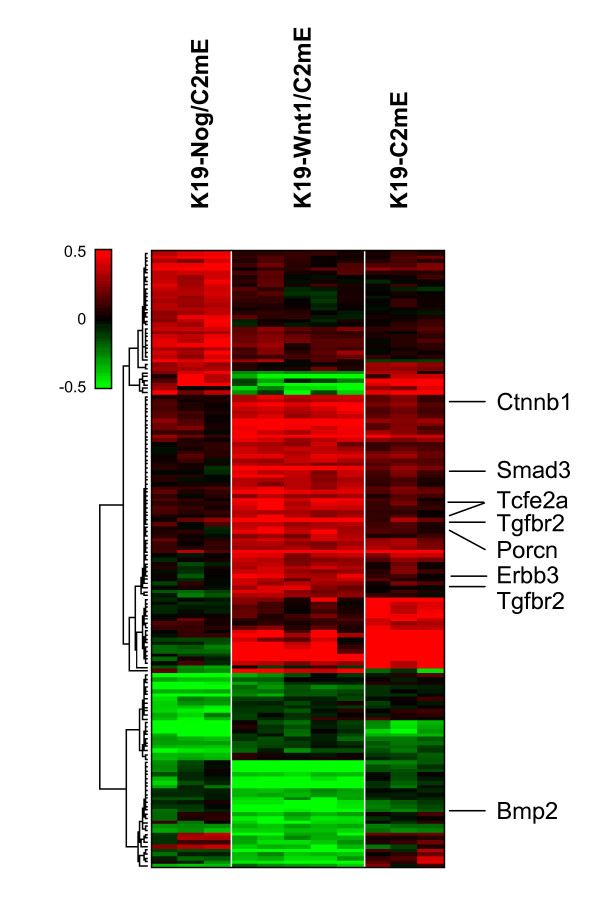
**Wnt/β-catenin regulatory genes are up-regulated in Wnt1/C2mE mice**. Clustered in rows are 155 probe sets which were differently regulated among three genotypes, *K19-C2mE*, *K19-Wnt1/C2mE*, and *K19-Nog/C2mE*, using ANOVA p-value threshold 0.001. Columns show mouse gastric sample grouped by genotype and genotypes are shown on top of the heatmap. Color scale is same as in Figure 1.

Tumors from three mouse models with PGE_2 _pathway activation show different histology. *K19-C2mE *develops hyperplasia with macrophage infiltration, whereas *K19-Wnt1/C2mE *develops dysplasia [17,18]. *K19-Nog/C2mE *develops hamartoma similar to human juvenile polyposis [19]. We next attempted to identify differentially expressed genes among the three mouse models which allowed us to assess the best-fit model among the three to study gastric intestinal-type cancer. With ANOVA p-value threshold of 0.001, we selected 155 genes which were differently regulated among the three groups. Few of these genes showed expression changes in the same direction between *K19-Wnt1/C2mE *and *K19-Nog/C2mE *(Figure 4). Wnt pathway genes *Porcn*, an acyltransferase required for Wnt protein secretion, β-catenin (*Ctnnb1*), and *Tcfe2a *(*TCF3 *in human) were overexpressed in *K19-Wnt1/C2mE *mice, but not in *K19-Nog/C2mE *(Figure 4). TGF-β/BMP pathway genes *Smad3 *and *Tgfbr2 *were also up-regulated and *Bmp2 *was down-regulated in *K19-Wnt1/C2mE *but not in *K19-Nog/C2mE*.

In *K19-Nog/C2mE *mice, some genes which promote tumorigenesis were up- or down-regulated, although they have not been reported in the downstream of BMP pathway. *ROCKII *was specifically up-regulated in *K19-Nog/C2mE*, and its overexpression is associated with progression in several types of cancers via modulating actin cytoskeleton organization. Down-regulated genes include *RAMP2 *and *PPARGC1A*, and their inactivation or under-expression was shown to contribute to lung cancer and hepatoma development respectively.

Since deregulation of Wnt pathway including *APC *or *CTNNB1 *mutation have been more frequently observed in intestinal-type compared with diffuse-type [23,24], the results indicated that *K19-Wnt1/C2mE *could offer a model that best-fits intestinal-type tumors among the three C2mE-related mice.

## Discussion

The present study indicated that human intestinal-type gastric cancers exhibited significant similarity to C2mE-related mice, especially to *K19-Wnt1/C2mE *mice by global expression profiling. The prediction of similar tumor type by global expression profile is consistent with the phenotypes of the transgenic mice. Accumulating evidence has indicated that inflammation level which is caused by the up-regulated expression/activity of *COX-2 *and *mPGES-1 *is severer in intestinal-type gastric cancer compared with diffuse-type one, although both types of tumors are related to *Helicobacter pylori *that are known to induce inflammation to the infected site [14,25-28]. This knowledge supports our observation that gastric tumors in C2mE-related mice in which PGE_2 _pathway is activated exhibit similarity to intestinal-type gastric tumors. In addition, activating and inactivating mutations in *CTNNB1 *and *APC *are more frequently observed in intestinal-type cancer. No *APC *LOH/mutation were observed in diffuse-type gastric cancer, whereas 60% were found in intestinal-type one [24,29,30]. Mutation in *CTNNB1 *was predominantly observed in intestinal-type one [13]. This is also concordant with our previous finding that *K19-Wnt1/C2mE *mice which only develop adenocarcinoma among the three C2mE-related mice activate down stream genes of Wnt/β-catenin pathway.

Usually, several types of transgenic mice for one tumor type are required to examine similarity in global expression profiling between mice tumor models and human ones, since the genes which were up- or down-regulated in each mice model were extracted compared to the average of all the examined tumor samples. With this approach, Lee *et al*. [31] analyzed gene expression data of seven mouse hepatocellular carcinomas (HCCs) including five GEMs with human HCCs to identify models that recapitulate human cancer or a type of human cancer, and found that some subclasses of human HCC mimic mice models in expression pattern. Hershkowitz *et al*. [32] also used the same normalization method, and found that characteristic expression patterns observed in human breast tumors were conserved in 13 mouse breast tumor models. Since the available data of expression profile for mouse gastric tumors are limited to our *K19-C2mE *and its compound mice, we took different strategy to assess the similarity of gastric tumors between the two species. Instead of using average of all samples in the dataset as a reference to calculate expression ratios, we normalized the mouse gastric data to average of wild-type samples. To compare our mice expression profiles with those of human gastric cancers, the gene signature to classify human intestinal- and diffuse-type gastric cancers was also modified from original one by normalizing the expression data to the average of normal gastric samples. This has allowed us to reveal that C2mE-related transgenic mice resemble human intestinal-type gastric tumors in expression profiling.

Comparison of gene expressions between mouse models showed that simultaneous induction of Wnt1 and PGE_2 _deregulated not only gene expression of *Ctnnb1 *and *Porcn *in Wnt signaling but also *Smad3 *and *Tgfbr2 *in TGF-β/BMP signaling. Given the crosstalk between TGF-β/BMP and Wnt pathways has been reported in multiple previous studies, the deregulated expression of the genes in the additional signaling pathways could be explained by positive and negative feedback to the pathways from the up-regulated Wnt signaling. For example, BMP signaling is known to suppress β-catenin activity in intestinal stem cells [33]. BMP signaling could be repressed in *K19-Wnt1/C2mE*, because *Bmp2 *expression was significantly down-regulated. Increase in *Smad3 *and *Tgfbr2 *might be resulted from the negative feedback by BMP signaling suppression, as demonstrated in a study on TGF-β induced fibrosis [34]. In contrast to *K19-Wnt1/C2mE *transgenic mice, expression changes of the Wnt pathway genes were not observed in *K19-C2mE *and *K19-Nog/C2mE *mice. It would be of great interest to further analyze the crosstalk of signaling pathways in the compound transgenic mice.

## Conclusions

Genetically engineered mouse (GEM) models provide useful tools to study mechanism of tumorigenesis, to validate a new target for drug development, and to find biomarkers. Advances in genetic engineering have allowed us to develop a variety of transgenic or knockout models of human diseases. The main question on using GEMs as disease models is whether the model recapitulates the human disease. We previously developed several gastric tumor transgenic mice in which prostaglandin E_2 _pathway is activated. Although we conducted detailed histological analysis with the transgenic mice, it remained elusive whether global molecular changes in the transgenic mice reproduce features of human gastric tumors or not. This report has provided initial evidence that *K19-C2mE *and their compound mice, *K19-Nog/C2mE*, *K19-Wnt1/C2mE*, show similarity to human gastric cancer, especially to intestinal-type one by the analysis of mRNA expression profile. Among others, extraction of up- or down-regulated genes specifically in *K19-Wnt1/C2mE *or *K19-Nog/C2mE *respectively inferred that *K19-Wnt1/C2mE *mice would provide best-fit mouse model for intestinal-type gastric tumors. These findings would potentially provide various benefits in our future studies including elucidation of gastric tumorigenesis and optimal therapeutic target identification.

## Methods

### Stomach tissue samples

Construction of transgenic mice have been described in our previous studies [17-19]. Briefly, the *K19-Wnt1 *and *K19-Nog *strains overexpress *Wnt1 *and *Nog *genes, respectively, specifically in the stomach. *K19-C2mE *overexpresses the *mPGES-1 *gene and *COX-2 *genes simultaneously and specifically in the stomach. *K19-Wnt1/C2mE *and *K19-Nog/C2mE *are compound transgenic mice with *K19-Wnt1 *and *K19-Nog*, respectively; both mouse strains have *K19-C2mE*. For expression profiling, three wild-type C57BL/6, five *K19-Wnt1*, three *K19-C2mE*, five *K19-Wnt1/C2mE*, two *K19-Nog*, and three *K19-Nog/C2mE *mice were used. All animals used in this study were female mice aged 18-65 weeks. The glandular stomach of each mouse was cut for microarray analysis. All animal studies were carried out in accordance with good animal practice as defined by the Institutional Animal Care and Use Committee (IACUC).

### Microarrays

GeneChip Mouse Genome 430 2.0 Arrays (Affymetrix, Inc.) were used to monitor the expression profiles of the gastric samples. Total RNA was prepared using the RNeasy Mini Kit (QIAGEN) after treatment with TRIzol (Invitrogen Corp.), and labeled cRNA was prepared using standard Affymetrix protocols. The signal intensities of the probe sets were normalized by the Affymetrix Power Tools RMA method implemented in Resolver software (Rosetta Biosoftware), and log ratio values to the average of wild-type samples were calculated for each sample by using Resolver. All the microarray data were deposited at Gene Expression Omnibus (GEO) under dataset accession no. GSE16902 [35].

### Public human microarray data

Human gastric cancer [20] and breast cancer [36] microarray data were retrieved from the online supplement in the Stanford Microarray Database [37]. The gastric cancer data includes 68 intestinal-type cancer, 13 diffuse-type cancer, and 15 normal gastric samples. The breast cancer data include 115 breast tumor and seven normal tissue samples. Human colon cancer data [38], including 100 colorectal cancer and five normal tissue samples, were retrieved from NCBI GEO under accession GSE5206. The Ann Arbor lung tumor dataset [39] including 86 lung adenocarcinomas and 10 non-neoplastic lung samples was obtained from the United States National Cancer Institute website [40]. Expression values were transformed to log10 (ratio to geometric averages of normal samples) in order to compare with mouse data.

### Intestinal vs. diffuse type signature genes

Human gastric tumor data from Chen *et al*. [20] were used to develop an intestinal vs. diffuse type classifier. We selected genes that met the following criteria: (1) t-test p-value < 0.001 between the two groups, (2) opposite changes in the average expression of signature genes in intestinal-type tumors and that of signature genes in diffuse-type tumors. The false discovery rate was estimated by the Benjamini and Hochberg method [41]. The tumor classes of mouse and human samples were predicted by linear discriminant analysis using the signature score defined by the following formula:

Signature score = (Average log ratio of genes up-regulated in intestinal-type tumors and down-regulated in diffuse-type tumors) - (Average log ratio of genes down-regulated in intestinal-type tumors and up-regulated in diffuse-type tumors)

### Combining mouse and human gene expression data

In order to combine mouse data with human gastric cancer microarray data, mouse and human data were re-ratioed to the geometric average of wild-type and normal samples, respectively. When there was more than one probe set for a gene in a microarray, the averaged expression ratios were used for the gene. Next, using only homologous genes that are represented in both arrays, we merged the mouse and human data sets into a single data set. The mouse microarray contains 45,037 probe sets, which correspond to 21,066 Entrez genes, and the human microarray contains 6,688 probes, which correspond to 4,463 Entrez genes. When they were merged, 4,094 homologous genes were identified.

### Statistical analysis

The hypergeometric test for Gene Ontology enrichment was performed using the Gene Set Annotator developed by Rosetta Inpharmatics [42]. For the other statistical analyses in this study, the MATLAB software (MathWorks Inc.) was used.

## Authors' contributions

MO and HK designed the research. HO constructed the transgenic animals and prepared the stomach tissue samples. HI analyzed the microarray data and wrote the manuscript. All authors read and approved the final manuscript.

## Supplementary Material

Additional file 1 **A list of intestinal type vs. diffuse type signature genes**. Sequence accession, gene symbol, and the average of log10 ratios in intestinal-type and in diffuse-type, respectively, are shown for each of the 122 cDNAs. Click here for file
